# Comparison of two data interpretation programs in automated blood cell counters

**DOI:** 10.1155/S1463924690000128

**Published:** 1990

**Authors:** Izumi Tsuda, Kazue Setoguchi, Noriyuki Tatsumi

**Affiliations:** Department of Clinical and Laboratory Medicine Osaka City University Medical School Asahimachi Abeno Osaka 545 Japan

## Abstract

Two data interpretation programs incorporated into two different automated blood cell counters were evaluated. The programs analysed size distribution histograms for screening for abnormal specimens. The messages given by the programs were ‘normal’, ‘abnormal’ and ‘suspect’. ‘Abnormal’ messages meant abnormalities in the values of the measured parameters. ‘Suspect’ messages were given when the histograms showed either a possibility of the existence of abnormal blood cells, or an abnormal level of such cells; re-examination by a manual method was necessary for such specimens. For reference, the manual differential was done. The false-negative rate for each kind of message was less than 10% and efficiency was about 90%. These programs were helpful in avoiding unnecessary manual observations in reducing the workload in a routine clinical laboratory.


Journal of Automatic Chemistry, Vol. 12, No. 3 (May–June 1990), pp. 104–107

### Comparison of two data interpretation programs in automated blood cell counters

**Izumi Tsuda*, Kazue Setoguchi and Noriyuki Tatsumi**

*Department of Clinical and Laboratory Medicine, Osaka City University Medical School, Asahimachi, Abeno, Osaka 545, Japan*

*Two data interpretation programs incorporated into two different automated blood cell counters were evaluated. The programs analysed size distribution histograms for screening for abnormal specimens. The messages given by the programs were 'normal', 'abnormal' and 'suspect'. 'Abnormal' messages meant abnormalities in the values of the measured parameters. 'Suspect' messages were given when the histograms showed either a possibility of the existence of abnormal blood cells, or an abnormal level of such cells; re-examination by a manual method was necessary for such specimens. For reference, the manual differential was done. The false-negative rate for each kind of message was less than 10% and efficiency was about 90%. These programs were helpful in avoiding unnecessary manual observations in reducing the workload in a routine clinical laboratory.*

### Introduction

Size distribution histograms and their parameters provided by blood cell counters reflect morphological specificities [1–6]. As the result of integration of information, data interpretation programs have been developed for blood cell counters. The interpretation programs alert the operator to samples with an abnormal count or an abnormal histogram. These programs remove the need for manual observation of the majority samples. This paper reports on the value of two different programs which used the same specimens from out-patients.

### Materials and methods

### Sample blood

Samples of venous blood treated with dipotassium ethylenediammine acid were randomly selected from samples obtained from out-patients ($N = 289$). For further evaluation of messages from the data interpretation programs, abnormal specimens from in-patients were also selected. The specimens were first analysed with an automated blood cell counter (STKR, Coulter Electronics, Inc., Hialeah, USA), and their morphology was microscopically observed with the Giemsa method. After these routine procedures the specimens were examined with two blood cell counters; both were based on the impedance principle and both contained computerized data interpretation programs.

### Data interpretation programs

Two data interpretation programs were evaluated; IR (Coulter; program A), and DI-1000 (Toa Medical Electronics, Kobe, Japan; program B). The definition and the number of messages shown by the two interpretation systems were different, so only some of the messages available with both were evaluated. The messages were divided into three categories: 'normal', 'abnormal' and 'suspect'. 'Normal' messages were shown for red blood cells, white blood cells, or platelets when both the histograms and the values obtained were normal. 'Abnormal' messages were definable by the operator and appeared when the values obtained exceeded present limits. The limits set by the manufacturers were used. The samples flagged as 'abnormal' could require re-examination, depending on laboratory protocol. The 'abnormal' messages evaluated were: 'granulopenia', 'granulocytosis', 'lymphopenia' and 'lymphocytosis'. 'Suspect' messages appeared when there was an abnormal distribution or an abnormal cell population, so microscopic review of stained smears was required to check for the abnormalities suggested. The programs for 'suspect' messages were defined by the manufacturers and could not be changed by the user. The 'suspect' messages alerted the operator to the existence of abnormal cells or to their increase and the following messages were included: 'eosinophils', 'basophils', 'blasts', 'atypical lymphocytes', and 'immature granulocytes' for white blood cells, and 'nucleated red blood cells' for red blood cells.

### Manual method

For reference, four experienced technologists working in the Osaka area analysed the specimens by the method recommended by the US National Committee for Clinical Laboratory Standards (NCCLS Document H20-T, [7]). The 'abnormal' messages given by the counters were compared with the results obtained from the manual method using the criteria for routine procedures in our laboratory. The positives by the manual method for 'suspect' messages were as follows: 6% or more eosinophils by the manual method for the message of 'eosinophils'; 3% or more basophils for 'basophils'; and 1% or more blasts, atypical lymphocytes, or immature granulocytes for the messages of 'blasts', 'atypical lymphocytes', or 'immature granulocytes', respectively.

### Evaluation

Sensitivity, specificity and efficiency were calculated for each message [8].

* Correspondence to Izumi Tsuda.

### Results

*'Abnormal' messages*

Table 1 shows the percentages of false positives (FPs) and false negatives (FNs), and also the sensitivity, specificity and efficiency of each kind of 'abnormal' message. Program A gave a lower FP rate and somewhat higher specificity and efficiency for 'lymphocytosis' compared to program B, and program B gave a lower FP rate and higher sensitivity than program A for 'granulopenia'. For other messages, similar results were obtained by both programs.

*'Suspect' messages (see table 2)*

With program A, the percentage of FPs for the messages of 'eosinophils', 'blasts', 'immature granulocytes' and 'nucleated red blood cells' was higher but the FN rates for these messages were lower than those with program B. The message of 'atypical lymphocytes' was shown more often by program B (60 versus 27), so the FP rate was higher and the FN rate was somewhat lower by that program than with program A.

*Percentages of true positives (TPs) and FNs for samples with abnormally large numbers of certain cells (see table 3)*

Samples with abnormally large numbers of certain cells detected by the manual method were selected. About half of them were assayed on both counters, but the rest were not. The FNs were divided into two groups, one with other 'suspect' messages for white blood cells, including messages not evaluated here, and the other without any 'suspect' messages, for samples found by the counter to have a normal histogram. The percentage of TPs became higher as the percentages of the cells in question increased.

### Discussion

The latest models of blood cells counters provide cell size distribution histograms and their analysed parameters, as well as a complete blood count. It is possible to identify abnormal leukocytes and some specific diseases if an abnormal histogram is found [1–6]. Data interpretation programs are based on the analysis of these histograms and are designed to alert the operator to the possible presence of abnormalities in the blood cells. Some researchers doubt the need for manual inspection of all samples sent to a laboratory and suggest reservation of this method for selected specimens with abnormal counts [4–6]. The interpretation programs were designed for this purpose.

Evaluation of the 'abnormal' messages by the two interpretation programs gave similar results. Precise and accurate determinations by automated blood cell counters were made and the correlation of the indices determined by the different kinds of counters is good [9, 10]. Thus, the results obtained were reasonable, and the slight differences in the counters probably arose from the three different sets of criteria used by the two manufacturers and by the authors.

When interpretation programs are used, the samples flagged with 'suspect' messages should be examined manually. For out-patients, when other messages not evaluated here were included, 10·4% of the samples had 'suspect' messages with program A, and 29·0% had 'suspect' messages with program B. For the total samples with both out-patients and in-patients, these percentages were slightly higher. The FN rate for each 'suspect' message was less than 10% with both programs, much lower than the FN rate by the manual method reported

*Table 1. Evaluation of 'abnormal' messages shown by programs A and B.*

| | | FP (%) | FN (%) | Sensitivity (%) $\frac{TP}{TP+FN}$ | Specificity (%) $\frac{TN}{TN+FP}$ | Efficiency (%) $\frac{TP+TN}{TP+FN+TN+FP}$ |
|---|---|---|---|---|---|---|
| Granulopenia | A | 5·2 (15/289) | 3·1 (9/289) | 30·8 (4/13) | 94·6 (261/276) | 91·7 (265/289) |
| | B | 2·8 (8/289) | 3·5 (10/289) | 16·7 (2/12) | 97·1 (269/277) | 93·8 (271/289) |
| Granulocytosis | A | 7·3 (21/289) | 0 (0/289) | 100·0 (5/5) | 92·6 (263/284) | 92·7 (268/289) |
| | B | 8·3 (24/289) | 0 (0/289) | 100·0 (5/5) | 91·5 (260/284) | 91·7 (265/289) |
| Lymphopenia | A | 12·5 (36/289) | 0 (0/289) | 100·0 (3/3) | 87·4 (250/286) | 87·5 (253/289) |
| | B | 13·8 (40/289) | 0 (0/289) | 100·0 (3/3) | 86·0 (246/286) | 86·2 (249/289) |
| Lymphocytosis | A | 7·6 (22/289) | 0·7 (2/289) | 0 (0/2) | 92·3 (265/287) | 91·7 (265/289) |
| | B | 12·8 (37/289) | 0·7 (2/289) | 33·3 (1/3) | 87·1 (249/286) | 86·5 (250/289) |

*Notes:* FP, false positive; FN, false negative; TP, true positive; and TN, true negative.

*Table 2. Evaluation of 'suspect' messages shown by programs A and B.*

| | | FP (%) | FN (%) | Sensitivity (%) $\frac{TP}{TP + FN}$ | Specificity (%) $\frac{TN}{TN + FP}$ | Efficiency (%) $\frac{TP + TN}{TP + FN + TN + FP}$ |
|---|---|---|---|---|---|---|
| Eosinophils | A | 8·7 | 5·9 | 22·7 | 90·6 | 85·5 |
| | | (25/289) | (17/289) | (5/22) | (242/267) | (247/289) |
| | B | 0·7 | 6·9 | 9·1 | 99·3 | 91·8 |
| | | (2/289) | (20/289) | (2/22) | (265/267) | (267/291) |
| Basophils | A | 0 | 0·7 | 0 | 100·0 | 99·3 |
| | | (0/289) | (2/289) | (0/2) | (287/287) | (287/289) |
| | B | 0 | 0·7 | 0 | 100·0 | 99·3 |
| | | (0/289) | (2/289) | (0/2) | (287/287) | (287/289) |
| Blasts | A | 8·3 | 0 | 100·0 | 91·6 | 91·7 |
| | | (24/289) | (0/289) | (2/2) | (263/287) | (265/289) |
| | B | 4·2 | 0·7 | 0 | 95·8 | 95·2 |
| | | (12/289) | (2/289) | (0/2) | (275/287) | (275/289) |
| Atypical lymphocytes | A | 9·0 | 1·7 | 16·7 | 90·8 | 89·3 |
| | | (26/289) | (5/289) | (1/6) | (257/283) | (258/289) |
| | B | 19·7 | 0·7 | 60·0 | 79.9 | 78·9 |
| | | (57/289) | (2/289) | (3/5) | (226/283) | (228/289) |
| Immature granulocytes | A | 9·3 | 0·4 | 75·0 | 90·5 | 90·3 |
| | | (27/289) | (1/289) | (3/4) | (258/285) | (261/289) |
| | B | 0·7 | 1·4 | 0 | 99·3 | 97·9 |
| | | (2/289) | (4/289) | (0/4) | (283/285) | (283/289) |
| Nucleated red blood cells | A | 1·0 | 0 | 100·0 | 99·0 | 99·0 |
| | | (3/289) | (0/289) | (1/1) | (285/288) | (285/289) |
| | B | 0 | 0·3 | 0 | 100·0 | 99·7 |
| | | (0/289) | (1/289) | (0/1) | (288/288) | (288/289) |

*Notes:* FP, false positive; FN, false negative; TP, true positive; and TN, true negative.

*Table 3. Evaluation of 'suspect' messages for samples with abnormally large numbers of certain cells.*

| | | Program A | | FN | | Program B | | FN | |
|---|---|---|---|---|---|---|---|---|---|
| | Percentage of the cells by manual method | *N* | TP | With other 'suspect' messages | Without any 'suspect' messages | *N* | TP | With other 'suspect' messages | Without any 'suspect' messages |
| Eosinophils | ≤ 6 but ≤ 10 | 33 | 6 (18·2) | 2 (6·1) | 25 (75·8) | 45 | 7 (15·6) | 18 (40·0) | 20 (44·4) |
| | < 10 but ≤ 15 | 12 | 4 (33·3) | 0 (0) | 8 (66·7) | 11 | 2 (18·2) | 5 (45·5) | 4 (36·4) |
| | < 15 | 8 | 4 (50·0) | 1 (12·5) | 3 (37·5) | 9 | 7 (77·8) | 2 (22·2) | 0 (0) |
| Basophils | ≤ 3 but ≤ 5 | 1 | 0 (0) | 1 (100·0) | 0 (0) | 6 | 0 (0) | 2 (33·3) | 4 (66·7) |
| | < 5 | 4 | 0 (0) | 2 (50·0) | 2 (50·0) | 6 | 0 (0) | 3 (50·0) | 3 (50·0) |
| Blasts | ≤ 1 but ≤ 3 | 5 | 2 (40·0) | 0 (0) | 3 (60·0) | 2 | 0 (0) | 1 (50·0) | 1 (50·0) |
| | < 3 but ≤ 10 | 0 | 0 (0) | 0 (0) | 0 (0) | 1 | 0 (0) | 0 (0) | 1 (100·0) |
| | < 10 | 6 | 6 (100·0) | 0 (0) | 0 (0) | 3 | 1 (33·3) | 1 (33·3) | 1 (33·3) |
| Immature granulocytes | ≤ 1 but ≤ 3 | 20 | 11 (55·0) | 2 (10·0) | 7 (35·0) | 21 | 1 (4·8) | 13 (61·9) | 7 (33·3) |
| | < 3 | 11 | 7 (63·6) | 0 (0) | 4 (36·4) | 19 | 2 (10·5) | 11 (57·9) | 6 (31·6) |
| Atypical lymphocytes | ≤ 1 but ≤ 3 | 21 | 4 (19·0) | 1 (4·8) | 16 (76·2) | 5 | 2 (40·0) | 1 (20·0) | 2 (40·0) |
| | < 3 | 5 | 3 (60·0) | 0 (0) | 2 (40·0) | 2 | 1 (50·0) | 0 (0) | 1 (50·0) |

*Notes:* *N*, number of samples with large numbers of cells found by the manual method; TP, true positive; and FN, false negative. Numbers in parentheses show percentages.

by Koepke *et al.* [11]. Thus, these programs would be useful in selecting those samples which should be sent for manual observation.

Samples with increased numbers of abnormal cells were examined to answer two questions: (1) whether the programs could accurately detect each abnormality; and (2) how many FNs would appear. The TP rate for each abnormality was not very high; for example, four out of eight were TPs for the samples with more than 15% eosinophils with program A; and one out of three was a TP for a sample with more than 10% blasts by program

B. This does not present a major problem in practice, however, because some kind of 'suspect' message will generally appear for abnormal samples.

Program A could detect each kind of abnormality somewhat more accurately than program B. On the other hand, it is also of practical importance to decrease the FN rate because if samples are not flagged with 'suspect' messages, then they will probably not be re-examined. In this respect, program B was superior to program A, although its FP rate was higher. One reason for this difference was that program B had more kinds of 'suspect' messages and showed them more often. The usefulness of the two programs evaluated was not very different; it depended on which false kind of result, FP or FN, is given more importance by the individual laboratory.

There were some differences between the two programs, but for screening abnormal samples for manual review, both programs were more useful than employing an abnormal count alone. With an appropriate protocol, it is possible to reduce the number of samples needing manual examination without increasing the FN rates. These systems should contribute substantially to saving both labour and time in clinical laboratories.

### References

1. Bates, J. E. and Bessman, J. D., *American Journal of Clinical Pathology*, **88** (1987), 314.

2. Bessman, J. D., Gardner, F. H. and Gilmer, P. R., *American Journal of Clinical Pathology*, **80** (1983), 242.

3. Bessman, J. D., Williams, L. C. and Gilmer, P. R., *American Journal of Clinical Pathology*, **78** (1982), 150.

4. Charache, S., Nelson, L., Keyer, E. and Metzger, P., *Archives of Internal Medicine*, **145** (1985), 1582.

5. Dutcher, T. F., *Blood Cells*, **11** (1985), 49.

6. Payne, B. A. and Pierre, R. V., *Laboratory Medicine*, **17** (1986), 517.

7. Tentative Standard H20-T, Leukocyte Differential Counting, National Committee for Clinical Laboratory Standards, Villanova, Pennsylvania (1984), Vol. 4, No. 11.

8. Mass, D. and Galen, R. S., *American Journal of Medical Technology*, **47** (1981), 965.

9. Carlson, D. A., Ito, R. K., Statland, B. E., Daigneault, R., DiPierro, R. and Horion, L., *American Journal of Clinical Pathology*, **86** (1986), 55.

10. Reardon, D. M., Hutchinson, D., Bradey, L. and Trowbridge, E. A., *Medical Laboratory Science*, **44** (1987), 320.

11. Koepke, J. A., Dotson, M. A. and Shifman, M. A., *Blood Cells*, **11** (1985), 173.

